# In Vitro Activity of Imipenem-Relebactam, Meropenem-Vaborbactam, Ceftazidime-Avibactam and Comparators on Carbapenem-Resistant Non-Carbapenemase-Producing Enterobacterales

**DOI:** 10.3390/antibiotics12010102

**Published:** 2023-01-06

**Authors:** Rémy A. Bonnin, Sandrine Bernabeu, Cécile Emeraud, Thierry Naas, Delphine Girlich, Agnès B. Jousset, Laurent Dortet

**Affiliations:** 1 Team “Resist” UMR1184 “Immunology of Viral, Auto-Immune, Hematological and Bacterial Diseases (IMVA-HB)”, INSERM, Faculty of Medicine, Paris-Saclay University, 94270 Le Kremlin-Bicêtre, France; 2 Associated French National Reference Center for Antibiotic Resistance: Carbapenemase-Producing Enterobacteriales, 94270 Le Kremlin-Bicêtre, France; 3 Department of Bacteriology-Hygiene, Assistance Publique des Hôpitaux de Paris, Bicêtre Hospital, 94270 Le Kremlin-Bicêtre, France

**Keywords:** inhibitors, extended-spectrum beta-lactamase, CRE, MIC

## Abstract

Background: Avibactam, relebactam and vaborbactam are β-lactamase inhibitors that proved their efficiency against KPC-producing Enterobacterales. Regarding their inhibitor activity towards Ambler’s class A extended spectrum β-lactamases (ESBL) and Ambler’s class C cephalosporinase (AmpC), they should be active on most of the carbapenem-resistant non-carbapenemase-producing Enterobacterales (CR non-CPE). Objectives: Determine the in vitro activity of ceftazidime-avibactam, imipenem-relebactam and meropenem-vaborbactam and comparators against CR non-CPE. Methods: MICs to ceftazidime/avibactam, imipenem/relebactam, meropenem/vaborbactam, but also temocillin, ceftolozane/tazobactam, ertapenem, colistin, eravacycline and tigecycline were determined by broth microdilution (ThermoFisher) on a collection of 284 CR non-CPE (inhibition zone diameter < 22 mm to meropenem). Whole genome sequencing was performed on 90 isolates to assess the genetic diversity as well as resistome. Results: According to EUCAST breakpoints, susceptibility rates of ceftazidime, imipenem, meropenem and ertapenem used at standard dose were 0.7%, 45.1%, 14.8% and 2.5%, respectively. Increased exposure of ceftazidime, imipenem and meropenem led to reach 3.5%, 68.3% and 67.7% susceptibility, respectively. Using the EUCAST clinical breakpoints, susceptibility rates of ceftazidime/avibactam, imipenem/relebactam and meropenem/vaborbactam were 88.4%, 81.0% and 80.6%, respectively. Susceptibility rates of temocillin, ceftolozane/tazobactam, tigecycline, eravacycline, and colistin were 0%, 4.6%, 27.8%, 54.9% and 90.1%. MICs distributions with and without the presence of the inhibitor demonstrated a better ability of avibactam and relebactam compared to vaborbactam to restore susceptibility to the associated β-lactam. Conclusions: This study demonstrated the in vitro efficacy of ceftazidime/avibactam, imipenem/relebactam and to a lesser extent meropenem/vaborbactam against CR non-CPE. Moreover, to test all β-lactams/β-lactamases inhibitors combinations without a priori for CRE, non-CPE is crucial since resistance to one of the β-lactam/β-lactamase inhibitor combinations does not predict resistance to another molecule, depending on the resistance mechanisms involved.

## 1. Introduction

Carbapenem resistance in Enterobacterales is increasingly reported worldwide [1]. Since the pipeline of new antimicrobials including new β-lactams remain very scarce, one hope resides in the introduction of new β-lactamase inhibitors for the treatment of infections caused by carbapenem-resistant Enterobactarales (CRE).

In Enterobacterales, carbapenem resistance (CR) is due to the production of a carbapenemase or to the (over)expression of broad-spectrum β-lactamases (ESBL or cephalosporinase) associated with weak permeability of the outer membrane (altered expression or inactivated porins) [2]. Among the carbapenemases identified in Enterobacterales, five are predominant, including Ambler class A KPC enzymes, metallo-β-lactamases (Ambler class B) of NDM, VIM and to a lesser extent IMP-type and Ambler class D OXA-48-like enzymes [2]. Carbapenem-resistant non-carbapenemase producing Enterobacterales (CR non-CPE) have been described. In *Klebsiella pneumoniae*, the role of two porins has been well documented. These two porins, namely OmpK35 and OmpK36, are involved in decreased susceptibility to carbapenems [3,4]. In *E. coli*, non-enzymatic resistance to carbapenems can also involve mutations in penicillin-binding protein 2 (PBP-2), which is the target of carbapenems [5]. In *Enterobacter cloacae* complex as well as for *Citrobacter freundii* complex, the role of derepression of cephalosporinase associated to decreased permeability in carbapenem resistance has been clearly demonstrated [6,7].

Recently, several β-lactam/β-lactamase inhibitor combinations have been marketed including ceftazidime/avibactam imipenem/relebactam meropenem/vaborbactam and cefolozane/tazobactam [8,9]. Relebactam and avibactam belong to diazabicyclooctanes and are marketed in combination with imipenem and ceftazidime, respectively [9]. Vaborbactam is a boronic acid derivative and is marketed in combination with meropenem. All these associations have been approved for the treatment of urinary tract infection, complicated intra-abdominal infections and ventilated acquired pneumonia. Relebactam and vaborbactam demonstrated efficient inhibition of Ambler class A enzymes, including KPC. Moderate inhibition of cephalosporinases (Ambler class C) and OXA-48-like enzymes (Ambler class D carbapenemases) (for relebactam only) and no inhibition of class B [10]. Avibactam possesses additional inhibition activity towards OXA-48-like carbapenemases (Ambler class D) [11]. Biochemical studies (measurement of IC_50_ and K_i_) confirmed the in vitro inhibition of class A and class C by relebactam and vaborbactam [12]. However, scarce data are available regarding the efficacy of these inhibitors against carbapenenem resistant non-carbapenemase-producing Enterobacterales (CR non-CPE).

In this study, the in vitro activities of ceftazidime-avibactam, imipenem-relebactam and meropenem-vaborbactam and comparators were tested against a collection of CR non-CPE received at the French National Reference Center (F-NRC).

## 2. Material and Methods

### 2.1. Strains Collection

A collection of *n* = 284 carbapenem-non-susceptible and non-carbapenemase-producing Enterobacterales (inhibition diameter < 22 mm for meropenem) was used in this study. Absence of carbapenemase production was assessed by the NG-Carba5 immunochromatographic assay (NG-Biotech, Guipry, France) and the Carba NP test as previously described [13,14]. All potential duplicates were discarded from the study. The collection was composed of *Klebsiella pneumoniae* (*n* = 145) *Klebsiella aerogenes* (*n* = 28), 11 *Klebsiella oxytoca* (*n* = 11), *Enterobacter cloacae* complex (*n* = 52), *Escherichia coli* (*n* = 32), *Citrobacter freundii* complex (*n* = 4), *Hafnia alvei* (*n* = 10) and *Serratia marcescens* (*n* = 1).

### 2.2. Determination of the Mechanism Responsible for Decreased Susceptibility to Carbapenems

As previously described for isolates having carbapenem susceptibility restored on Mueller–Hinton agar supplemented with 200 mg/L cloxacillin, the overexpression of a cephalosporinase (chromosome encoded or plasmid acquired) was suspected [15]. The ESBL production was evidenced by double disc synergy performed on classical MH agar but also on a cloxacillin-supplemented medium to detect the overexpression of a cephalosporinase (chromosome encoded or plasmid acquired), plus the co-expression of an ESBL. As previously described, reduced susceptibility to moxalactam (inhibition zone diameter of 23 mm for 30 mg of moxalactam) was used as a marker of decreased outer-membrane permeability [16].

### 2.3. Antimicrobial Susceptibility Testing and MIC Determination

MICs were determined using broth microdilution using precoated plate (Thermofischer, Les Ulis, France). MICs were interpreted using EUCAST breakpoints as updated in 2022 [17,18].

### 2.4. Whole Genome Sequencing

A total of *n* = 90 representative isolates were sequenced to assess their exact resistome and clonal relationship. Whole genome sequencing was performed using Illumina’s Nextseq 500 at PIBNET sequencing platform (Institut Pasteur, Paris, France). All genomes were assembled and analyzed as previously described [19] and natural β-lactamases were sought using Resfinder v4.1 [20].

## 3. Results

### 3.1. Analysis of the Strains Collection

The 284 CR non-CPE included in this study were phenotypically classified in six distinct groups:

(1) ESBL producers coupled with membrane permeability defect (named ESBL) (*n* = 123)
(2) Isolates overproducing their intrinsic cephalosporinase coupled membrane permeability defect (named CASE) (*n* = 68)
(3) A mix of intrinsic cephalosporinase overproduction associated with ESBL production and membrane permeability defect (named ESBL + CASE) (*n* = 36)
(4) Isolates producing an acquired cephalosporinase associated with membrane permeability defect (named aCASE) (*n* = 14)
(5) *Klebsiella pneumoniae* or *Klebsiella oxytoca* overproducing their intrinsic penicillinase SHV-like or OXY-like (named Hyper-SHV or Hyper-OXY) (*n* = 16 and *n* = 4, respectively)
(6) Unclassified phenotype (named Unknown) (*n* = 25) (Supplementary Materials Table S1).

Among the 284 CRE non-CPE included in this study, 90 were sequenced using NGS. Resistome analysis of these strains revealed that CTX-M-15 was the most prevalent ESBL (*n* = 43) produced among ESBL or ESBL + CASE producers (*n* = 52), followed by SHV-12 (*n* = 2) and by CTX-M-1, CTX-M-3, CTX-M-8, CTX-M-33, CTX-M-71, OXA-35, and SHV-2 (*n* = 1 each). Regarding the *n* = 14 aCASE producers, DHA-1 was the most prevalent acquired cephalosporinase (*n* = 4), followed by CMY-2 (*n* = 2), CMY-146 (*n* = 2), CMY-42 (*n* = 1) and DHA-7 (*n* = 1). Analysis of clonal relationship indicated a wide variety of ST indicating that the collection was diverse.

### 3.2. Susceptibility to Meropenem-Vaborbactam Compared to Meropenem

**Figure 1:**
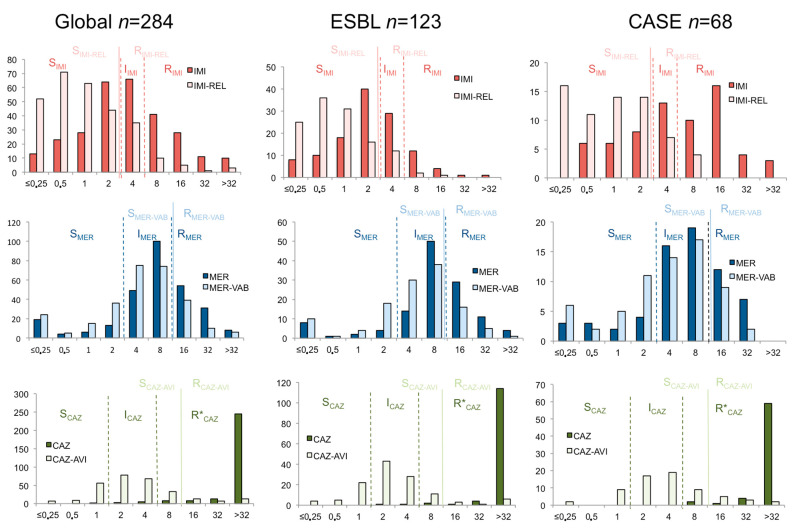
MIC distributions of imipenem. imipenem/relebactam meropenem, meropenem/vaborbactam, ceftazidime and ceftazidime/avibactam on a collection of 284 carbapenem-resistant non-carbapenemase-producing Enterobacterales. The global collection (*n* = 284) of CRE non-CPE was divided by resistance mechanisms in three categories (Global) (*n* = 284), ESBL-producing isolates (ESBL) (*n* = 123) and deregulated cephalosporinases (CASE) (*n* = 68). EUCAST breakpoints are indicated by dashed lines (β-lactam alone) and continuous lines (β-lactam/inhibitor combination) dashed lines.

According to EUCAST guidelines, clinical breakpoints for meropenem are at ≤2 and >8 mg/L whereas meropenem/vaborbactam is at ≤8 mg/L [17]. Since the high dosage of meropenem was included in the meropenem/vaborbactam combination, the high dosage regimen of meropenem must be compared to the standard dosage of meropenem/vaborbactam to assess the efficiency of vaborbactam to restore meropenem efficacy. In our collection of Enterobacterales with meropenem inhibition diameter < 22 mm. A total of 14.8% of the tested isolates remained susceptible at standard dose (meropenem MIC ≤ 2 mg/L) and 67.3% at high dosage of meropenem (meropenem MIC ≤ 8 mg/L) whereas 80.6% of the strains were categorized as susceptible to meropenem-vaborbactam combination (meropenem/vaborbactam MIC ≤ 8 mg/L) (Table 1 and Figure 1). The addition of vaborbactam allowed a decrease of the MIC_50_ and the MIC_90_ of meropenem by a one 2-fold dilution (Table 1). Regarding ESBL producers (*n* = 123), susceptibility rates to meropenem at standard dosage, meropenem at high dosage and meropenem/vaborbactam were of 12.2%. 64.2% and 82.1%, respectively (Figure 1). Regarding cephalosporinase producers (*n* = 68), susceptibility rates to meropenem at standard dosage, meropenem at high dosage and meropenem/vaborbactam were of 18.2%, 69.1% and 83.3%, respectively (Figure 1). Overall, vaborbactam allowed restoring meropenem susceptibility in 17.9% of the ESBL producers and 14.2% of the cephalosporinase producers.

### 3.3. Susceptibility to Imipenem-Relebactam Compared to Imipenem

According to EUCAST guidelines, clinical breakpoints for imipenem and imipenem-relebactam combination are as follows: ≤2 mg/L and >4 mg/L for imipenem and ≤2 mg/L for imipenem/relebactam. Since the standard dosage of imipenem was present in the imipenem/relebactam combination, the standard dosage regimen of imipenem must be compared to the standard dosage of imipenem/relebactam to assess the efficiency of relebactam to restore imipenem efficacy. Overall, susceptibility rates of 45.1% (imipenem MIC ≤ 2 mg/L) and 68.3% (imipenem MIC ≤ 4 mg/L) were observed for imipenem at standard and high dosage, respectively. Regarding imipenem/relebactam combination, 81.0% of isolates were susceptible to the combination (imipenem-relebactam MIC ≤ 2 mg/L) (Table 1). The addition of relebactam allowed the MIC_50_ and the MIC_90_ of imipenem of two 2-fold dilution to decrease (Table 1). Regarding ESBL producers (*n* = 123), susceptibility rates to imipenem at standard dosage, imipenem at high dosage and imipenem/relebactam were 61.8%. 95.1% and 87.8%, respectively (Figure 1). Regarding cephalosporinase producers (*n* = 68), susceptibility rates to imipenem at standard dosage, imipenem at high dosage and imipenem/relebactam were 30.3%. 65.2% and 83.3%, respectively (Figure 1). Overall, relebactam allowed restoring imipenem susceptibility in 26.0% of the ESBL producers and 53.0% of the cephalosporinase producers.

### 3.4. Susceptibility to Ceftazidime-Avibactam Compared to Ceftazidime

It is difficult to directly compare susceptibility rate of ceftazidime and ceftazidime-avibactam combination. Indeed, clinical breakpoints were different between ceftazidime alone and ceftazidime combined with avibactam. According to EUCAST, clinical breakpoints of ceftazidime are at ≤1 mg/L and >4 mg/L whereas ceftazidime-avibactam combination displays only one clinical breakpoint at ≤8 mg/L. In our collection, susceptibility rates for ceftazidime used at standard and high dosage were of 0.7% and 3.5%, respectively (Table 1). The susceptibility rate of ceftazidime/avibactam was of 88.4% (MIC ≤ 8 mg/L). However, 25.3% and 76.8% of the tested isolates possessed ceftazidime-avibactam MIC ≤ 1 mg/L and ≤4 mg/L, respectively (Figure 1). The addition of avibactam allowed for a decrease in the MIC_50_ and the MIC_90_ of ceftazidime of more than four and two 2-fold dilution, respectively (Table 1).

### 3.5. Susceptibility to Temocillin, Ceftolozane/Tazobactam, Ertapenem, Colistin, Eravacycline and Tigecycline

In this collection of CRE non-CPE, temocillin, ceftolozane/tazobactam and ertapenem exhibited very low susceptibility rates with 10.9%. 4.6% and 2.5%, respectively. The efficacy of cyclines remained moderate with susceptibility rates of 27.8% and 54.9% for tigecycline and eravacycline, respectively (Table 2). The most potent compound in vitro remains colistin with 90.1% susceptibility.

### 3.6. Susceptibility Testing without a Priori for CRE Non-CPE

One of the key issues for the treatment of infections is the early adaption of the treatment. In the case of CRE non-CPE, it seemed crucial to test all β-lactam/β-lactamase inhibitor combinations without a priori. Indeed, as shown in Table 3, resistance to one of the β-lactams/β-lactamases inhibitor combinations does not predict resistance to another molecule. Ceftazidime-avibactam seemed to be the most efficient β-lactam/β-lactamase inhibitor combination for the treatment of CRE non-CPE that is also resistant to all carbapenems (imipenem, meropenem and ertapenem), to imipenem/relebactam and to meropenenem/vaborbactam. However, among the 33 CRE-non-CPE isolates that were categorized as resistant to ceftazidime-avibactam (MIC > 8 mg/L), imipenem/relebactam and meropenem/vaborbactam still displayed 54.5% and 48.5% susceptibility, respectively. Of note, contrary to meropenem/vaborbactam combination in which the meropenem dosage corresponds to the high dosage of meropenem (2 g), the dosage of imipenem included in the imipenem/relebactam combination corresponds to the standard dosage of imipenem (0.5 g). Accordingly, despite that it is not recommended by the approvals delivered by the European Medicine Agency nor the Food and Drug Administration, it might be possible to increase the dosage of imipenem by adding a standard dose of imipenem to a standard dose of imipenem/relebactam. Thus, it might make sense to align the breakpoint of the “high dosage” of imipenem/relebactam to the breakpoint corresponding to the high dosage of imipenem at 4 mg/L (instead of 2 mg/L, which corresponds to the breakpoint of standard dosage of imipenem and imipenem/relabactam). Using this potential adaptation of the dosage (and of the breakpoint), imipenem/relebactam would be as efficient as ceftazidime/avibactam in the treatment of CRE non-CPE. Again, among the 19 isolates that remained resistant to this “high dosage” of imipenem/relebactam (MIC > 4 mg/L), ceftazidime/avibactam and meropenem/vaborbactam still displayed 78.9% and 21.1% susceptibility, respectively.

## 4. Discussion

The performances of imipenem/relebactam and meropenem/vaborbactam, as well as ceftazidime/avibactam, have extensively been established for the treatment of carbapenemase producers. In this study, we tested these β-lactam/β-lactamase inhibitors against a collection of *n* = 284 CR non-CPE. Among this collection, the main mechanism of carbapenem-resistance in *K. pneumoniae* was the production of an ESBL associated with decreased membrane permeability. This mechanism corresponds to the global epidemiology of CR non-CPE *K. pneumoniae* in other countries [21,22]. In *C. freundii* complex and *E. cloacae* complex, the main mechanism corresponds to the over-production of the natural cephalosporinase associated with decreased membrane permeability as described previously [6,7]. Of note, some variants of natural cephalosporinase can exhibit a weak carbapenemase activity in *Enterobacter kobei* [23].

Sequencing of *n* = 90 isolates confirmed that the main ESBL corresponds to *bla*_CTX-M-15_ as it is globally observed [24]. Other ESBLs correspond *bla*_SHV-12_, *bla*_CTX-M-1_, *bla*_CTX-M-3_, *bla*_CTX-M-8_, *bla*_CTX-M-33_, *bla*_CTX-M-71_, *bla*_OXA-35_, and *bla*_SHV-2_. The main cephalosporinases were *bla*_CMY-_ or *bla*_ACT-_like that corresponds to the natural cephalosporinase of *C. freundii* complex and *E. cloacae* complex, respectively. The clonal relationship was alos assessed using whole genome analysis. This analysis showed a wide genetic diversity and demonstrated that our collection includes a variety of resistance mechanisms and clones.

These three tested β-lactams/β-lactamases inhibitor combinations have been developed for the treatment of KPC producers. Unfortunately, none of these inhibitors (avibactam, relebactam and vaborbactam) is able to inhibit metallo-β-lactamases. Finally, only the combination of avibactam with ceftazidime possesses a significant activity towards OXA-48-like carbapenemase producers [18]. The effect of ceftazidime/avibactam on OXA-48-producing Enterobacterales was mainly due to the fact that OXA-48 is unable to hydrolyze ceftazidime [25]. Thus, avibactam inhibits associated β-lactamase rather than direct inhibition of OXA-48. Despite the resistance to carbapenems in Enterobacterales, mostly involve the association of membrane with decreased permeability and expanded spectrum β-lactamases that could be inhibited by avibactam, relebactam and vaborbactam (ESBLs or cephalosporinases), still few data are available regarding the efficacy of these inhibitors against CRE non-CPE with the exception of a recent paper in which 45 CR non-CPE were also tested [26].

The direct comparison of efficacy of avibactam, relebactam and vaborbactam is difficult since the breakpoints as well as the associated molecules are different. Breakpoints for imipenem/relebactam combination follow a standard regimen of imipenem being at 2 mg/L whereas breakpoints for meropenem/vaborbactam follow a high dosage of meropenem of 8 mg/L. The case of ceftazidime/avibactam is even trickier since the cut-off value for ceftazdime/avibactam combination is higher than the breakpoint used for the high dose of ceftazidime only (being at 4 mg/L for ceftazidime alone and 8 mg/L for the ceftazdime/avibactam combination). The probability of target attainment using Monte Carlo simulations alone could not explain these differences since these data are similar for carbapenems and carbapenem/inhibitor combinations [17,27,28]. However, focusing on these CRE non-CPEs with meropenem inhibition diameter < 22 mm, we identified a significant effect of the addition of avibactam, relebactam and to a lesser extent vaborbactam to restore the activity of their respective associated β-lactam. In our collection, MICs distributions, with and without the presence of the inhibitor, demonstrated a better ability of avibactam and relebactam compared to vaborbactam to restore susceptibility to the associated β-lactam.

Relebactam allowed imipenem to restore its efficacy at standard dose (MIC ≤ 2 mg/L) in 35.9% of the isolates, while avibactam helped to restore efficacy of ceftazidime at standard dose (MICs ≤ 1 mg/L); for 25.0% of the tested strains and vaborbactam restored efficacy of meropenem at standard dose (MIC ≤ 2 mg/L) for only 13.4% of the tested isolates. Similar results were obtained if a high dose of the associated molecule was used; Relebactam allowed to restore imipenem susceptibility at a high dose (MIC ≤ 4 mg/L) in 25.0% of the isolates, while avibactam helped to restore susceptibility to ceftazidime at high dose (MICs ≤ 4 mg/L) for 73.3% of the tested strains; and vaborbactam allowed to restore meropenem susceptibility at a high dose (MIC ≤ 8 mg/L) for only 13.3% of the tested isolates. It should be acknowledged that the selection of strains using the meropenem inhibition diameter might induce a bias. However, as shown in Table 3, for isolates that are resistant to all carbapenems, the inhibition activity of avibactam, relebactam seemed to remain better than varborbactam at reaching susceptibility.

In this study, we demonstrated the crucial role of testing all β-lactams/β-lactamases inhibitors combinations without *a priori* for CRE non-CPE since resistance to one of the β-lactams/β-lactamases inhibitor combinations does not predict resistance to another. We also highlighted the fact that the percentage of resistance/susceptibility alone does not reflect the efficacy of an inhibitor since the clinical breakpoints can be different with or without inhibitors.

## Figures and Tables

**Table 1:** MICs distribution for β-lactams and β-lactams/β-lactamases inhibitors combinations.

		MIC Distribution (mg/L)													
	*n*	≤0.25	0.5	1	2	4	8	16	32	>32	MIC_50_	MIC_90_	% S	% I	% R
Temocillin	284	0	0	0	1	0	3	27	88	165	>32	>32	0.0	14.1	85.9
Ceftazidime	284	0	0	2	3	5	8	8	13	245	>32	>32	0.7	2.8	96.5
Ceftazidime/avibactam	284	7	9	56	78	68	33	13	7	13	2	16	88.4		11.6
Ceftolozane/tazobactam	284	0	0	7	6	13	22	34	24	178	>32	>32	4.6		95.4
Ertapenem	284	5	2	2	11	11	15	38	72	128	32	>32	2.5		97.5
Imipenem	284	13	23	28	64	66	41	28	11	10	4	16	45.1	23.2	31.7
Imipenem/relebactam	284	52	71	63	44	35	10	5	1	3	1	4	81.0		19.0
Meropenem	284	19	4	6	13	49	100	54	31	8	8	32	14.8	52.5	32.7
Meropenem/vaborbactam	284	24	5	15	36	75	74	39	10	6	4	16	80.6		19.4

Red: Resistant; Green: susceptible according to EUCAST breakpoints.

**Table 2:** MICs distribution for colistin and cyclines.

		MIC Distribution (mg/L)														
	N	≤0.25	0.5	1	2	4	8	16	32	64	128	>128	MIC_50_	MIC_90_	% S	% R
Colistin	284	17	176	54	9	1	12	5	4	0	5	1	0.5	4	90.1	9.9
Eravacycline	284	73	83	42	46	35	5 ^a^	ND ^b^	ND ^b^	ND ^b^	ND ^b^	ND ^b^	0.5	4	54.9	96.5
Tigecycline	284	20	59	85	63	43	14 ^a^	ND ^b^	ND ^b^	ND ^b^	ND ^b^	ND ^b^	1	4	27.8	72.2

^a^ ≥8 mg/L; ^b^ ND: Not determined. Red: Resistant; Green: susceptible according to EUCAST breakpoints.

**Table 3:** Susceptibility rates to β-lactam, β-lactamase/inhibitors according to the dose of β-lactam.

Phenotypes	N	% of Susceptibility											
		CAZ (High Dose) (≤4 mg/L)	CZT (≤2 mg/L)	ETP (≤0.5 mg/L)	IMP (Standard Dose) (≤2 mg/L)	IMP (High Dose) (≤4 mg/L)	IMP-REL (≤2 mg/L)	^a^ IMP-REL (High Dose) (≤4 mg/L)	MEM (Standard Dose) (≤2 mg/L)	MEM (High Dose) (≤8 mg/L)	MEM-VAB (Standard Dose) (≤8 mg/L)	CAZ-AVI (Standard Dose) (≤8 mg/L)	COL (≤2 mg/L)
ETP R (>0.5 mg/L)	277	3.2	3.6	0.0	43.7	67.5	80.5	93.1	12.6	66.4	80.1	88.1	90.3
IMI R (>4 mg/L)	90	3.3	3.3	0.0	0.0	0.0	46.7	78.9	0.0	37.8	53.3	80.0	91.1
MEM R (>8 mg/L)	93	3.2	2.2	0.0	12.9	39.8	55.9	82.8	0.0	0.0	43.0	78.5	86.0
IMI R (>4 mg/L) + MEM R (>8 mg/L) + ETP R (>0.5 mg/L)	56	5.4	3.6	0.0	0.0	0.0	30.4	71.4	0.0	0.0	26.8	75.0	87.5
CZA R (>8 mg/L)	33	0.0	0.0	0.0	18.2	45.5	54.5	87.9	3.0	39.4	48.5	0.0	84.8
IMP-REL R (>2 mg/L)	54	7.4	5.6	0.0	0.0	11.1	0.0	64.8	0.0	24.1	35.2	72.2	90.7
IMP-REL high dose R (>4 mg/L)	19	15.8	10.5	0.0	0.0	0.0	0.0	0.0	0.0	15.8	21.1	78.9	89.5
MEM-VAB R (>8 mg/L)	55	5.5	3.6	0.0	0.0	23.6	36.4	72.7	0.0	0.0	0.0	69.1	89.1

CAZ, ceftazidime; CZT, ceftolozane/tazobactam; IMP, imipenem; IMP-REL, imipenem/relebactam; MEM, meropenem; MEM-VAB, meropenem/vaborbactam; CAZ-AVI, ceftazidime-avibactam; COL, colistin. ^a^ the high dosage of imipenem/relebactam might be obtained by doubling the dosage of imipenem/relebactam or by mixing a standard dose of imipenem and a standard dose of imipenem/relebactam. Of note, this dosage is not recommended by the approvals delivered by the European Medicine Agency nor the Food and Drug Administration.

## Data Availability

All data are in our lab on demand.

## Supplementary Materials

The following supporting information can be downloaded at: https://www.mdpi.com/article/10.3390/antibiotics12010102/s1, Table S1: MICs and phenotype/genotype of carbapenem non-susceptible isolates used in this study.
